# Noise Reduction and Image Quality Improvement of Low Dose and Ultra Low Dose Brain Perfusion CT by HYPR-LR Processing

**DOI:** 10.1371/journal.pone.0017098

**Published:** 2011-02-11

**Authors:** Radko Krissak, Charles A. Mistretta, Thomas Henzler, Anastasios Chatzikonstantinou, Johann Scharf, Stefan O. Schoenberg, Christian Fink

**Affiliations:** 1 Institute of Clinical Radiology and Nuclear Medicine, University Medical Center Mannheim, Medical Faculty Mannheim, Heidelberg University, Heidelberg, Germany; 2 Department of Medical Physics, University of Wisconsin, Madison, Wisconsin, United States of America; 3 Department of Neurology, University Medical Center Mannheim, Medical Faculty Mannheim, Heidelberg University, Heidelberg, Germany; 4 Department of Neuroradiology, University Medical Center Mannheim, Medical Faculty Mannheim, Heidelberg University, Heidelberg, Germany; University of Texas, M.D. Anderson Cancer Center, United States of America

## Abstract

**Purpose:**

To evaluate image quality and signal characteristics of brain perfusion CT (BPCT) obtained by low-dose (LD) and ultra-low-dose (ULD) protocols with and without post-processing by highly constrained back-projection (HYPR)–local reconstruction (LR) technique.

**Methods and Materials:**

Simultaneous BPCTs were acquired in 8 patients on a dual-source-CT by applying LD (80 kV,200 mAs,14×1.2 mm) on tube A and ULD (80 kV,30 mAs,14×1.2 mm) on tube B. Image data from both tubes was reconstructed with identical parameters and post-processed using the HYPR-LR. Correlation coefficients between mean and maximum (MAX) attenuation values within corresponding ROIs, area under attenuation curve (AUC), and signal to noise ratio (SNR) of brain parenchyma were assessed. Subjective image quality was assessed on a 5-point scale by two blinded observers (1:excellent, 5:non-diagnostic).

**Results:**

Radiation dose of ULD was more than six times lower compared to LD. SNR was improved by HYPR: ULD vs. ULD+HYPR: 1.9±0.3 vs. 8.4±1.7, LD vs. LD+HYPR: 5.0±0.7 vs. 13.4±2.4 (both p<0.0001). There was a good correlation between the original datasets and the HYPR-LR post-processed datasets: r = 0.848 for ULD and ULD+HYPR and r = 0.933 for LD and LD+HYPR (p<0.0001 for both). The mean values of the HYPR-LR post-processed ULD dataset correlated better with the standard LD dataset (r = 0.672) than unprocessed ULD (r = 0.542), but both correlations were significant (p<0.0001). There was no significant difference in AUC or MAX. Image quality was rated excellent (1.3) in LD+HYPR and non-diagnostic (5.0) in ULD. LD and ULD+HYPR images had moderate image quality (3.3 and 2.7).

**Conclusion:**

SNR and image quality of ULD-BPCT can be improved to a level similar to LD-BPCT when using HYPR-LR without distorting attenuation measurements. This can be used to substantially reduce radiation dose. Alternatively, LD images can be improved by HYPR-LR to higher diagnostic quality.

## Introduction

Cranial CT is still the most used imaging test for the diagnostic workup of stroke since CT is available on a 24 h routine base in most medical centers. Because unenhanced CT may underestimate both the severity of ischemia and the spatial extent of hypoperfusion, brain perfusion CT (BPCT) has been proposed for improving the detection of ischemic stroke and evaluating the extent and severity of hypoperfusion as this has major impact on the indication of thrombolytic therapy [1], [2], [3].

Recently, the very small but finite cancer risk associated with radiation by CT has attracted greater public attention [4], [5]. Consensus has been reached that the ‘as low as reasonably achievable’ (ALARA) principle should be applied more consistently. Comprehensive stroke imaging on a 64 slice multi-detector CT (MDCT) including a noncontrast scan, perfusion CT and CT angiography may result in a total dose of up to 9.5 mSv with possible organ doses of the brain of up to 490 mGy [6]. Although critical doses for organ damage are not reached, physicians need to be aware of possible radiation induced sequelae particularly in repetitive examinations [6]. For this reason, BPCT is usually performed as a low dose protocol using low milliampere and low kilovolt settings with a radiation dose lower than that of a standard unenhanced CT of the brain [7]. The low dose protocol is unfortunately resulting in a higher amount of image noise, which is compensated by using thick-slices and/or reduced matrix reconstructions and/or spatial smoothing, all at cost of lowering spatial resolution [2], [7].

The CT formulation of HighlY constrained back PRojection (HYPR) imaging was introduced recently and recommended for use in time-resolved CT angiography [8]. The HYPR algorithm was originally developed for application to contrast-enhanced magnetic resonance angiography to shorten the data acquisition time and improve temporal resolution [9]. When this new post-processing technique is applied to CT imaging, the improved noise properties provide the potential to considerably reduce the radiation dose in each time frame without significantly compromising the image quality. Two different approaches can be used to lower the delivered radiation dose using HYPR methods: the first method is to reduce the tube current, and the second method is to acquire fewer projection views [8].

The purpose of this feasibility study was to prospectively evaluate the image quality and signal characteristics of an ultra low dose (ULD, more than 6-fold dose reduction) BPCT protocol post-processed with the HYPR local reconstruction (LR) algorithm compared to a simultaneously acquired standard low dose (LD) BPCT. Furthermore, the image quality and signal characteristics of HYPR-LR-post-processed standard LD BPCT datasets was evaluated. To our knowledge, this is the first patient study evaluating HYPR-LR for perfusion CT.

## Materials and Methods

### Patients

In this feasibility study, simultaneous LD und ULD BPCT acquisitions were performed in 8 patients (2 women, 6 men; mean age 61±15 years; age range 35–88 years) who were already hospitalized and had neurological symptoms suspicious of subacute ischemic stroke. Emergency patients eligible for thrombolysis were not included in this feasibility study. None of the investigated patients had contraindication against contrast-enhanced CT such as renal insufficiency or allergy to a contrast material. The study protocol was approved by the institutional review board of the University Medical Center Mannheim. Explanations about the nature of the imaging procedure were provided to each patient and written informed consent was obtained.

### CT technique

All exams were performed on a 64-channel dual-source CT scanner (Somatom Definition, Siemens Medical Solutions, Forchheim, Germany). The system is equipped with two X-ray tubes and two corresponding detectors which are orientated in the gantry with an angular offset of 90°. One detector array (corresponding to tube A) provides a field of view (FOV) of 50 cm, while the other detector array (corresponding to tube B) is restricted to a FOV of 26 cm [10], [11]. Both X-ray tubes can be operated at different kV and mA settings, allowing the simultaneous acquisition of a ultra low dose scan and a standard reference scan in one examination without compromising the image quality or patient comfort [10]. Thus it creates an opportunity for a direct comparison of different CT protocols.

The patients were well centered in the scanner gantry to ensure that the entire head is covered by the smaller field-of-view of the second tube detector array. A diagnostic native spiral CT of the brain (120 kV, 420 mAs, 40×0.6 mm collimation, 0.55 pitch, 5 mm slice thickness reconstruction) was performed before the BPCT examinations. BPCT was performed with 80 kV tube voltage for tube A and B with a standard of reference LD protocol [12], [13] on tube A and ULD protocol on tube B. The LD and the ULD BPCT datasets were acquired simultaneously in one scan using tube currents of 200 mA on tube A and 30 mA on tube B. Automatic tube current modulation (CARE Dose 4D) was turned off during the acquisition. The detector collimation, rotation time and table feed were 14×1.2 mm, 330 ms and 0 mm. The imaging range was positioned above the orbits. Continuous cine scanning was performed during a total scanning time of 60 seconds, with a scan rate of 1 image per second. 50 ml of Iomeprol 400 (Imeron 400, Bracco Imaging S.p.A., Milan, Italy) were injected using a power injector with an injection rate of 5 ml/s and followed by a saline chaser of 20 ml using the same flow rate. The scan was started with a delay of 4 s. A separate dataset for each tube with a slice thickness of 5 mm and a reconstruction increment of 4 mm using a soft tissue kernel (B30f) was calculated. The volume CT dose index (CTDI_vol_) and dose length product (DLP) were recorded for each acquisition to estimate the radiation dose.

### HYPR-LR post-processing

**Figure 1:**
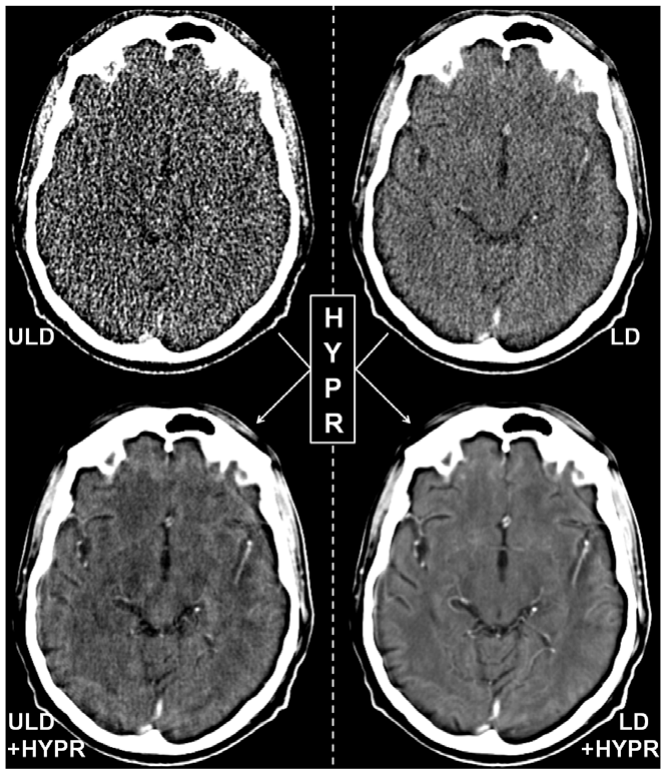
Example images of ultra low dose (ULD), HYPR-LR-post-processed ultra low dose (ULD+HYPR), low dose (LD) and HYPR-LR-post-processed low dose (HYPR+LD) brain perfusion CT of a 58-years old male patient with no pathology. The images are all reconstructed with the same slice thickness (5 mm) and presented with identical window settings.

After image reconstruction of the two data sets from both tubes (80 kV/200 mAs and 80 kV/30 mAs), the DICOM-images were further processed by the HYPR-LR noise-reduction algorithm using Matlab (Mathworks, Natick, Massachusetts, USA). An example of images before and after HYPR-LR-post-processing is presented in Figure 1. The functionality of HYPR was initially described by Mistretta et al. [9], further development to HYPR-LR was described by Johnson et al. [14]. Supanich et al. introduced the CT formulation for HYPR [8]. The functionality of the algorithm is described in detail in the mentioned original publications. In brief, the HYPR-LR algorithm consists of two fundamental steps used to combine spatial and temporal information. The first step is to reconstruct a low-noise-variance composite image C(x, y, z) using information all time frames. In the composite image, there is no dynamic information about the image object, but the signal-to-noise level is superior to that of the individual time frame. In the second step, the composite image is used to constrain the reconstruction of an individual low-radiation-dose time frame. The final image at a time frame denoted by I(x, y, z; t) is given by the product of the composite image and a weighting image, w(x, y, z; t), as follows:



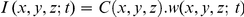



The image noise variance in the final image only weakly depends on the noise properties of the individual time frame. This property is the key to implementing radiation dose reduction in time-resolved imaging.

### Data analysis

First a diagnosis was assessed in consensus by two radiologists, a board certified neuroradiologist with 15 years of experience in CT imaging and a third year resident, using all existing data including laboratory tests, optionally performed MRI or biopsy findings. Then, subjective image quality of the LD, ULD and the corresponding HYPR-LR-post-processed datasets was assessed on a 5 point scale (1: excellent, 5: non-diagnostic) in consensus by two radiologists blinded to the dataset acquisition/post-processing technique, a board certified radiologist with 10 years of experience in CT imaging and a fourth year resident.

Normalized Cerebral Blood Flow (CBF), Cerebral Blood Volume (CBV), Mean Transit Time (MTT) and Time to Peak (TTP) color-coded maps were automatically created using IB Neuro (Imaging Biometrics, Elm Grove, Wisconsin, USA) after defining a reference arterial input function in three arterial timepoints in different arterial segments spatially separated by at least 2 cm and normal appearing white matter within a region of interest drawn by the user (board certified radiologist with 10 years of experience in CT). The utilized software does not use reduced matrix reconstructions or spatial smoothing, the images are left noisy. The calculated color-coded images were not used for diagnosis. Only evaluation of subjective diagnostic quality was performed. For each slice location, a rectangular region of interest (ROI) of 11×11 pixels was drawn into the brain parenchyma in the temporal lobe in the first image of every LD time series and was copied to all subsequent images of the same time series as well as to all corresponding images of the original ULD and the respective HYPR-LR post-processed time series. The temporal lobe was chosen in all patients for the purpose of standardization, the size and shape of the ROI were chosen arbitrarily to cover an area of at least 100 pixels.

### Statistical analysis

Pearson correlation coefficients were calculated to assess the correlations within all ROIs between the mean attenuation values in the original and the post-processed datasets (LD vs. LD +HYPR, ULD vs. ULD+HYPR), between those in the LD and the ULD+HYPR datasets as well as between those in the LD and the ULD datasets. Bland-Altman analysis was also applied to evaluate the agreement between the mean attenuation values of the above listed datasets [15]. Area under the attenuation curve (AUC), maximum attenuation, minimum attenuation in the brain parenchyma were assessed in each time series. The signal to noise ratio (SNR) of the brain parenchyma in every time series was calculated as mean SNR of all images in the time series. Paired Student's t-test was calculated to compare the AUC, maximum attenuation, minimum attenuation and SNR values between the datasets.

## Results

In the consensus reading, three out of 8 patients had perfusion abnormalities within the examined volume. These included a chronic right frontal infarction, a chronic right posterior boundary zone infarction, and an enhancing lung cancer metastasis adjacent to the right thalamus as well as a chronic left frontal infarction. The diagnosis of normal pressure hydrocephalus without focal perfusion abnormalities was made in one patient. No acute stroke was diagnosed.

**Figure 2:**
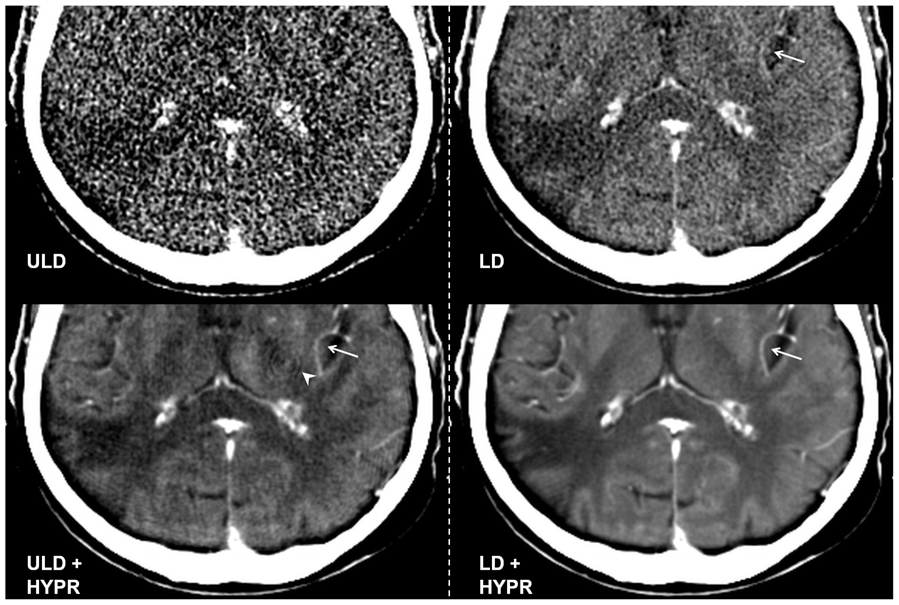
Ultra Low Dose (ULD), HYPR-LR-post-processed ULD (ULD+HYPR), Low Dose (LD) and HYPR-LR-post-processed LD (LD+HYPR) brain perfusion CT of a 68-year old male patient with a chronic infarction in the posterior territory of the right MCA (detail view). All images acquired at the same time point (last image of the 60 s time series). The ULD image was rated non-diagnostic (5), the infarction is not clearly visualized, details like small vessels (arrow in the other images) can not be clearly identified due to high image noise. Ring artifacts, which were present but not recognizable in the noisy ULD image are recognizable after HYPR post-processing (arrowhead in ULD + HYPR). Despite the artifacts, the subjective image quality was rated equal in ULD+HYPR and LD and images (both classified as 3). Excellent subjective image quality (classified as 1) in the LD+HYPR image with good differentiation between gray and white matter.

**Figure 3:**
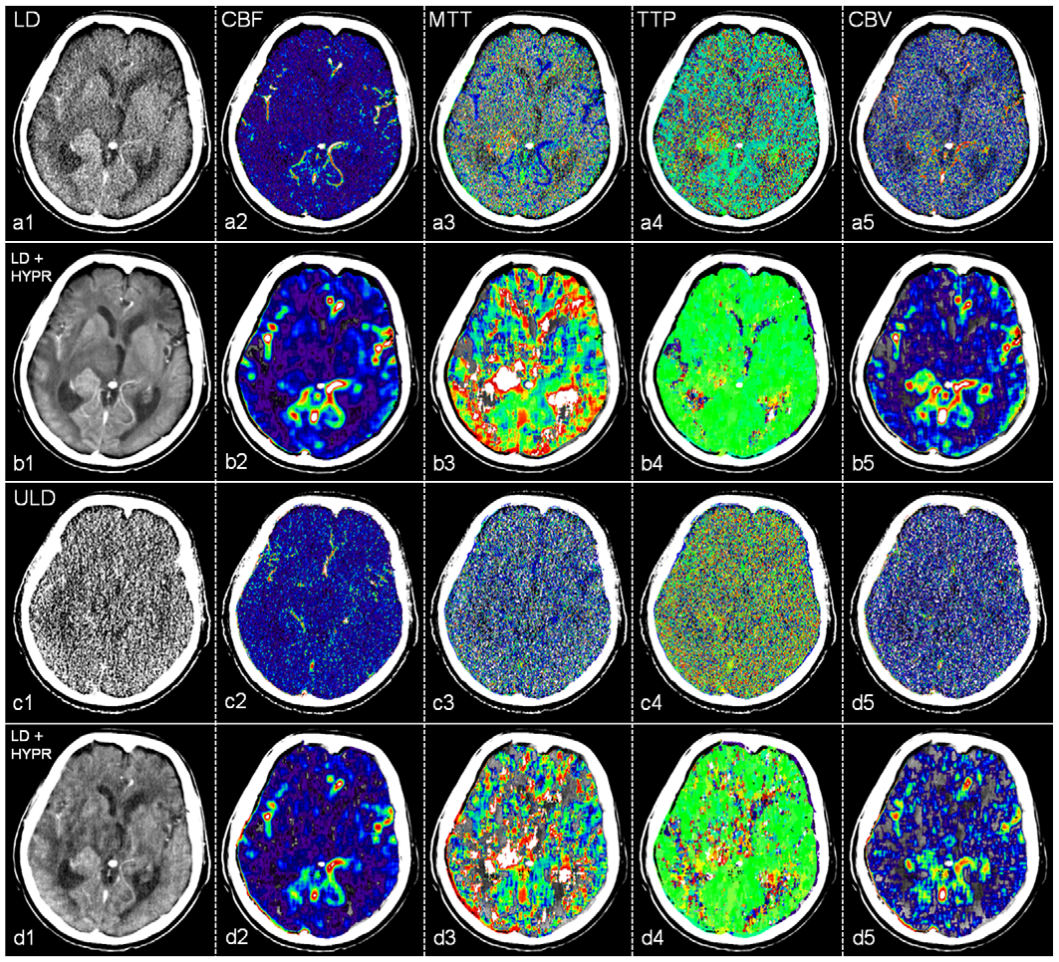
Low dose (LD, a), HYPR-LR-post-processed low dose (LD+HYPR, b), ultra low dose (ULD, c) and HYPR-LR-post-processed ultra low dose (ULD+HYPR, d) brain perfusion CT of a 75-year old male patient with a lung cancer metastasis adjacent to the right thalamus and chronic left frontal infarction. Last image of the 60 s time series (1) and the normalized cerebral blood flow (CBF, 2), mean transit time (MTT, 3), time to peak (TTP, 4), cerebral blood volume (CBV, 5) maps. The utilized software does not use reduced matrix reconstructions or spatial smoothing, the images are left noisy. The pathology is recognizable in a, b and d with excellent subjective image quality and low noise in b. No diagnosis possible in c.

The radiation dose of ULD BPCT (mean DLP 47.1±0.4 mGy×cm, range 47–48 mGy×cm; mean CDTI_VOL_ 28.09±0.05 mGy, range 28.06–28.12 mGy) was more than six times lower than that of LD BPCT (mean DLP 362.1±0.4 mGy×cm, range 362–363 mGy×cm; mean CDTI_VOL_ 215.35±0.38 mGy, range 215.14–216.10 mGy). The subjective image quality was rated excellent (1.3±0.5, range 1–2) in the LD+HYPR and non-diagnostic (5.0±0.2, range 4–5) in ULD images. LD and ULD+HYPR images both were rated as having moderate image quality (3.3±0.5, range 3–4 and 2.7±0.7, range 2–4). Details like small vessels, which were present but not recognizable in the noisy ULD BPCT images were clearly recognizable after HYPR-LR post-processing, but also ring artifacts resulting from low X-ray detector input were visualized (Figure 2). No sufficient visual analysis of the normalized CBF, CBV, MTT and TTP perfusion maps was possible in the unprocessed ULD BPCT datasets (Figure 3).

**Figure 4:**
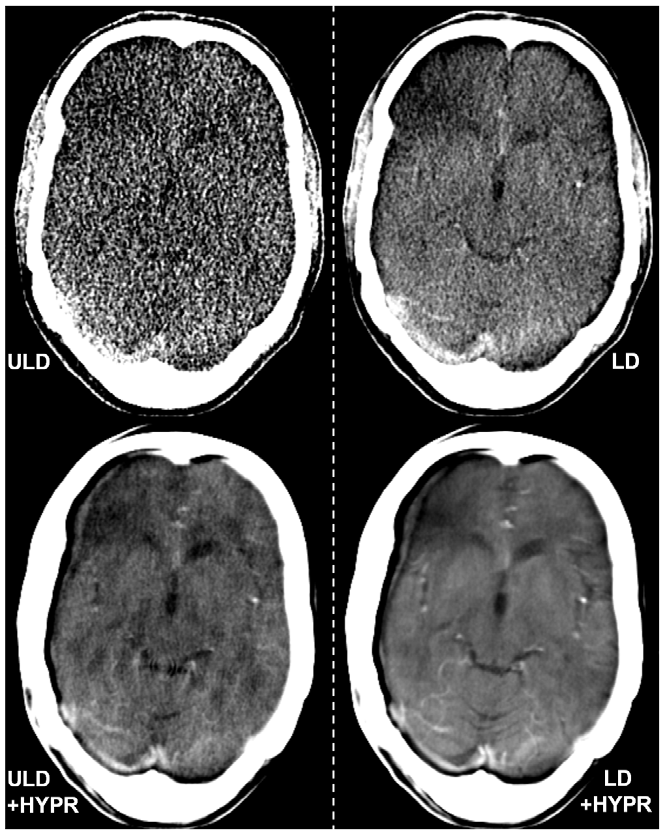
Ultra low dose (ULD), HYPR-LR-post-processed ultra low dose (ULD+HYPR), low dose (LD) and HYPR-LR-post-processed low dose (HYPR+LD) brain perfusion CT of a 35-years old patient with no pathology. This patient has slightly moved his head several times starting after 8 seconds of the data acquisition. As the HYPR-algorithm is using information of all time frames in the composite image for the calculation of the individual images, this resulted in an artifact visible in all HYPR-LR-post-processed images of this patient with a double contour of the skull and the brain on the right side and a frontal right hypodensity. The frontal right hypodensity was also visible in some non-post-processed images. The subjective image quality of the LD+HYPR image (rated 3) was still preferred to LD and ULD+HYPR (both rated 4). The ULD image was subjectively non-diagnostic (5). In the case of motion artifacts image registration might further improve image quality if used before the HYPR-LR algorithm is applied.

One patient has slightly moved his head several times starting 8 seconds after the initiation of the data acquisition. As the HYPR-algorithm is using information of all time frames in the composite image for the calculation of the individual images, this resulted in an artifact visible in all HYPR-LR post-processed images of this patient (Figure 4). Because all individual frames in a time series are used to calculate the CBF, CBV, MTT and TTP maps, this artifact was also equally present in the non-HYPR-processed color-coded perfusion maps.

**Figure 5:**
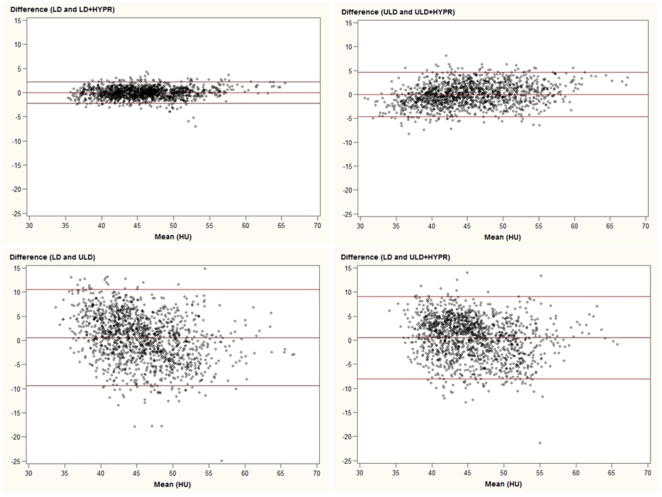
Bland-Altman plots describing the relationship between the mean attenuation values (1440 measurements) of the brain parenchyma in ultra low dose (ULD), HYPR-LR-post-processed ultra low dose (ULD+HYPR), low dose (LD) and HYPR-LR-post-processed low dose (HYPR+LD) brain perfusion CT.

**Figure 6:**
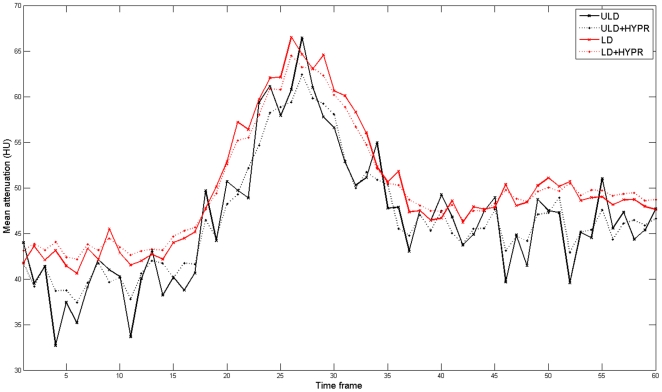
Mean attenuation values in a corresponding 60 s time series of the ultra low dose (ULD), the HYPR-LR-post-processed ultra low dose (ULD+HYPR), the low dose (LD) and the HYPR-LR-post-processed low dose (HYPR+LD) images.

There was a good correlation between the mean attenuation values of the original datasets and the HYPR-LR-post-processed datasets for ULD vs. ULD+HYPR and LD vs. LD+HYPR, respectively (r = 0.848 and r = 0.933, p<0.0001). The mean attenuation values of the HYPR-LR-post-processed ULD dataset showed a higher correlation with the standard LD dataset than with the unprocessed ULD dataset (r = 0.672 vs. 0.542), but both correlations were significant (p<0.0001). The correlation coefficients with 95% confidence intervals are summarized in Table 1. The agreement between the mean attenuation values of the datasets is also visualized in Bland-Altman plots in Figure 5. There was no significant difference in the calculated AUC and in the maximum attenuation values. There was a significant difference between the minimum attenuation values between the ULD and the LD datasets (36±5 HU vs. 40±3 HU, p = 0.001). However, there was no significant difference in minimum attenuation values between the ULD+HYPR dataset and the LD dataset (38±4 vs. 40±3 HU, p = 0.13). Example attenuation curves of the corresponding time series are provided in Figure 6. SNR could be significantly improved by HYPR-LR compared to the original datasets: ULD vs. ULD+HYPR: 1.9±0.3 vs. 8.4±1.7 (p<0.0001), LD vs. LD+HYPR: 5.0±0.7 vs. 13.4±2.4 (p<0.0001). The SNR of ULD+HYPR was also significantly higher than that of LD (p<0.001). The values are summarized in Table 2.

**Table 1:** Correlation of mean attenuation between original and post-processed data sets.

	Correlation coefficient	95% confidence interval	p-value
ULD and ULD+HYPR	0.848	0.760–0.905	<0.0001
LD and LD+HYPR	0.933	0.892–0.959	<0.0001
LD and ULD+HYPR	0.672	0.520–0.765	<0.0001
LD and ULD	0.542	0.358–0.699	<0.0001

**Table 2:** Subjective image quality scores and and quantitative attenuation measurements of LD and ULD and HYPR-LR post-processed BPCT data sets.

	Subjective image quality score	Maximum attenuation in time series (HU)	Minimum attenuation in time series (HU)	Area under attenuation curve in time series	SNR
**ULD,** mean ± SD (range)	5.0±0.2 (4–5)	57±6 (49–71)	36±5 (30–46)	2724±263 (2241–3210)	1.93±0.26 (1.40–2.32)
**ULD + HYPR,** mean ± SD (range)	2.7±0.7 (2–4)	56±7 (44–68)	38±4 (31–47)	2724±263 (2241–3210)	8.39±1.67 (5.08–11.32)
**LD,** mean ± SD (range)	3.3±0.5 (3–4)	55±6 (44–67)	40±3 (35–47)	2767±210 (2341–3157)	4.96±0.74 (3.77–6.69)
**LD + HYPR,** mean ± SD (range)	1.3±0.5 (1–2)	54±6 (44–65)	41±3 (36–47)	2758±211 (2341–3157)	13.36±2.36 (8.92–17.07)
p-value **ULD** vs. **ULD + HYPR**		0.33	0.07	0.99	<0.0001
p-value **LD** vs. **LD + HYPR**		0.63	0.35	0.89	<0.0001
p-value **LD** vs. **ULD + HYPR**		0.67	0.13	0.53	<0.0001
p-value **ULD** vs. **LD**		0.15	0.001	0.53	<0.0001

Note: Subjective quality score are reported as mean values.

## Discussion

Although BPCT is already routinely performed in low dose technique [12], [13], further dose reduction is desirable for every CT examination. Depending on the clinical situation serial BPCT examinations may be performed which result in a local radiation dose accumulation with radiation-induced tissue damage such as temporary hair loss as previously reported by a group performing serial examinations with a 4 row MDCT and DSA [16]. New technologic advances in CT (e.g. wider detector coverage, periodic spiral scans) make it possible to perform perfusion CT of the entire brain[17], [18]. Using the current generation of scanners covering up to 16 cm, even a higher cumulative radiation dose may occur in such patients. Radiologists should be aware that a cumulative or multiplier effect of radiation exposure from multiple diagnostic techniques may be present and should always look for new methods for lowering the radiation dose. In this feasibility patient study we evaluated the HYPR-LR post-processing algorithm, which can significantly increase the SNR in a time resolved series and thus could possibly help to further reduce radiation dose in BPCT exams [8]. After more than 6-fold radiation dose reduction by simply lowering the tube current, we observed a good correlation between the mean measurement values in LD and the ULD (more than six fold dose reduction), but a high level of noise in the ULD images which did not allow sufficient calculation of brain perfusion maps. After post-processing the ULD dataset by the HYPR-LR-algorithm, the SNR has improved significantly by an average factor of more than 4. The correlation factor between the standard LD and ULD+HYPR was higher than between the standard LD und ULD. The Bland-Altman plots also demonstrate a better agreement between the mean attenuation of the brain parenchyma in the standard LD and the HYPR-LR post-processed ULD images compared to LD and ULD. The fact that there was no significant difference in maximum attenuation but significant difference in minimum attenuation in a time series between LD and ULD can be explained by higher SNR after contrast enhancement. Minimum values are measured in the first native time frames with low SNR, maximum values are measured after iodine enhancement, which is responsible for higher SNR in both datasets. The increase of SNR is proportionally higher in the ULD dataset, leading to a better match between the maximum attenuation values in a time series. Higher SNR after HYPR-LR-post-processing also reduces the difference between ULD and LD for the minimum attenuation values.

A sufficient brain perfusion analysis is possible after a standard LD acquisition, but mostly at cost of lowering spatial resolution by using thick-slices and/or reduced matrix reconstructions and/or spatial smoothing [2], [12]. In our study, the SNR in the LD datasets could be improved by an average factor of 2.7 after HYPR-LR-post-processing, resulting in excellent subjective image quality. In cases where image quality or spatial resolution is preferred over radiation dose reduction, a LD acquisition with HYPR-LR-post-processing could be alternatively performed. One of major concerns in CT image post-processing is to ensure that the measurement values are not significantly modified. The high correlation coefficients and the good agreement in the Bland-Altman plots between the original and the post-processed datasets demonstrate reliable attenuation measurement values in the brain parenchyma after HYPR-LR post-processing.

The potential for reduction of radiation dose using HYPR-LR in time-resolved CT exams was previously investigated by Supanich et al.[8]. Both, mathematical simulations and in vivo canine studies, demonstrated the potential of a 6-8 -fold dose reduction. No patients studies were available for the evaluation of HYPR-LR in CT. In our feasibility study, we could validate a more than 6-fold dose reduction in patients. The average SNR of ULD+HYPR was about 1.7 times higher than that of standard LD, the subjective quality of ULD+HYPR was also slightly better compared to LD (2.7 vs. 3.3). This indicates that there is still a potential of further dose reduction. In a study performed on renal perfusion CT in pigs, a 10-fold dose reduction was evaluated without loss of accuracy. The image quality of the one-tenth dose images could be improved to be near that of the routine dose images in pigs by using the HYPR-LR noise-reduction algorithm [19]. In contrast to BPCT, which is accepted in the clinical routine, renal perfusion CT is mostly performed for research purposes. For this reason we decided to evaluate HYPR-LR for BPCT in humans. Ring artifacts caused by the combination of 3rd generation CT geometry and low X-ray detector input[20] were observed in the ULD and ULD+HYPR images in our study. Such artifacts were not described in the animal studies mentioned above. This might be explained by the non-central position of the examined small animal organ inside the CT gantry. These artifacts could limit a further dose reduction with the current CT generation.

The head movement of one patient in our study has created motion artifacts observed in all HYPR-LR-post-processed images of this patient. The HYPR-algorithm itself does not have a mechanism to account for the motion induced blurring artifacts. Especially in neuro-applications, accidental motion is typically isolated to a few time frames, and an image registration step can be used to correct the mis-registration before the HYPR technique is applied.

A limitation of our feasibility study is that patients with acute infarction were not examined. Subjectively high image quality of HYPR-LR-post-processed images with good differentiation between gray and white matter, good correlation between the measurement values, and visualization of chronic infarctions and metastasis in HYPR-LR-post- processed images in our feasibility study indicate similar results in acute stroke. A small patient number in our feasibility study does not allow a definitive evaluation of the potential of the HYPR-LR algorithm in BPCT, but the results justify for a further evaluation of the algorithm in a larger patient group.

In conclusion, SNR and image quality of ultra low dose BPCT can be improved to a level similar to low dose BPCT when using the HYPR-LR algorithm without distorting the attenuation measurements. This can be used to reduce the radiation dose by a factor of six compared to a standard low dose protocol. Alternatively, low dose BPCT images can be improved by HYPR-LR to a higher diagnostic quality.
